# Symbiont switching and trophic mode shifts in Orchidaceae

**DOI:** 10.1111/nph.17414

**Published:** 2021-06-01

**Authors:** Deyi Wang, Hans Jacquemyn, Sofia I. F. Gomes, Rutger A. Vos, Vincent S. F. T. Merckx

**Affiliations:** ^1^ Naturalis Biodiversity Center Leiden 2332 AA the Netherlands; ^2^ Institute of Biology Leiden University Leiden 2333 BE the Netherlands; ^3^ Department of Biology, Plant Conservation and Population Biology KU Leuven Kasteelpark Arenberg 31, Heverlee Leuven 3001 Belgium; ^4^ Department of Evolutionary and Population Biology Institute for Biodiversity and Ecosystem Dynamics University of Amsterdam Amsterdam 1098 XH the Netherlands

**Keywords:** ancestral state reconstruction, mixotrophy, mycoheterotrophy, orchid mycorrhiza, phylogenetic correlation, symbiont switching

## Abstract

- Mycorrhizal fungi are central to the biology of land plants. However, to what extent mycorrhizal shifts – broad evolutionary transitions in root‐associated fungal symbionts – are related to changes in plant trophic modes remains poorly understood.
- We built a comprehensive DNA dataset of Orchidaceae fungal symbionts and a dated plant molecular phylogeny to test the hypothesis that shifts in orchid trophic modes follow a stepwise pattern, from autotrophy over partial mycoheterotrophy (mixotrophy) to full mycoheterotrophy, and that these shifts are accompanied by switches in fungal symbionts.
- We estimate that at least 17 independent shifts from autotrophy towards full mycoheterotrophy occurred in orchids, mostly through an intermediate state of partial mycoheterotrophy. A wide range of fungal partners was inferred to occur in the roots of the common ancestor of this family, including ‘rhizoctonias’, ectomycorrhizal, and wood‐ or litter‐decaying saprotrophic fungi. Phylogenetic hypothesis tests further show that associations with ectomycorrhizal or saprotrophic fungi were most likely a prerequisite for evolutionary shifts towards full mycoheterotrophy.
- We show that shifts in trophic mode often coincided with switches in fungal symbionts, suggesting that the loss of photosynthesis selects for different fungal communities in orchids. We conclude that changes in symbiotic associations and ecophysiological traits are tightly correlated throughout the diversification of orchids.

## Introduction

The history of evolution and biodiversity is fundamentally a history of the evolution of species interactions (Margulis, 1991; Thompson, 1999, 2005). Many of the major events in the diversification of life can be traced back to the appearance of novel species interactions (Szathmáry & Smith, 1995). As such, the colonization and subsequent domination of land by plants – a fundamental turning point in the evolutionary history of the earth – was probably facilitated by new interactions with mutualistic symbiotic fungi (‘mycorrhizas’) that promote plant growth by facilitating the acquisition of essential nutrients (e.g. phosphorus, nitrogen and sometimes carbon) (Pirozynski & Malloch, 1975; Selosse & Le Tacon, 1998; Field *et al.,* 2015; Martin *et al.,* 2017; Feijen *et al.,* 2018). Today, the symbiosis with mycorrhizal fungi is found in over 90% of extant species of all major lineages of land plants, except for mosses, and involves 40 000–50 000 species of three different fungal phyla (Brundrett & Tedersoo, 2018; Tedersoo *et al.,* 2020). Based on the morphology of the interaction and the identity of the interacting plants and fungi, four major mycorrhizal types can be distinguished: arbuscular mycorrhiza, ectomycorrhiza, ericoid mycorrhiza and orchid mycorrhiza (Smith & Read, 2008; van der Heijden *et al.,* 2015; Brundrett & Tedersoo, 2018).

Interestingly, phylogenetic mapping of these four mycorrhizal types has shown that they are evolutionary‐conserved and that transitions between mycorrhizal types are relatively rare among land plants, fueling questions about what drives these switches (Feijen *et al.,* 2018; Werner *et al.,* 2018). Recent studies have suggested that the composition of mycorrhizal fungal communities differs between plant nutritional modes, ranging from an exclusively autotrophic to a fully mycoheterotrophic mode of life, in which photosynthesis has been replaced by the uptake of carbon from root‐associated fungi (Yagame *et al.,* 2016; Jacquemyn & Merckx, 2019). This raises the hypothesis that shifts in trophic modes are correlated with switches in symbiotic associations in land plants.

Orchids are particularly relevant to the investigation of this hypothesis in more detail as they form unique mycorrhizas with basidiomycete and ascomycete fungi, known as orchid mycorrhizas (OrM) (van der Heijden *et al.,* 2015; Brundrett & Tedersoo, 2018). Previous research has suggested that OrM probably evolved from an ancestor with arbuscular mycorrhizal fungi, in which the gain of OrM fungi is explained by pathogenic infection (Yukawa *et al.,* 2009; Rasmussen & Rasmussen, 2014). The evolution of OrM has been regarded as one of the major drivers for the evolutionary success of the Orchidaceae (Dressler, 2005; Rasmussen & Rasmussen, 2014; Chase *et al.,* 2015; Givnish *et al.,* 2015; Jacquemyn *et al.,* 2017a). Moreover, unlike the majority of green plants, orchids rely on carbon from OrM fungi for the germination of their dust seeds, a phenomenon known as ‘initial mycoheterotrophy’ (Leake, 1994; Rasmussen, 1995; Merckx, 2013). While most species likely become fully autotrophic as adults, several partially mycoheterotrophic species retain the ability to obtain carbon from their mycorrhizal fungi to compensate for photosynthesis (also termed mixotrophy; Selosse & Roy, 2009), and more than 250 fully mycoheterotrophic species have completely replaced their photosynthetic capacity by the uptake of fungal carbon (Jacquemyn & Merckx, 2019). Although these trophic modes have also evolved outside the Orchidaceae, no other plant family displays such a high frequency of shifts in trophic modes (Merckx, 2013; Jacquemyn & Merckx, 2019).

Traditionally, orchid mycorrhizas have been considered to consist primarily, if not only, of members of the ‘rhizoctonias’ complex, which comprises taxa from three distinct fungal families: Tulasnellaceae, Ceratobasidiaceae and Serendipitaceae (Smith & Read, 2008; Dearnaley *et al.,* 2012), and this association has been regarded as the ancestral state of the family (Yukawa *et al.,* 2009; Dearnaley *et al.,* 2012; Weiß *et al.,* 2016). However, more recently, other saprotrophic and ectomycorrhizal (ECM) fungal lineages have been found to form mycorrhizal associations with orchids (Bidartondo *et al.,* 2004; Dearnaley *et al.,* 2012; Ogura‐Tsujita *et al.,* 2021). The number and composition of fungal taxa associating with a single orchid are variable and depend on the phylogenetic relatedness of the orchids (Shefferson *et al.,* 2010; Jacquemyn *et al.,* 2011; Martos *et al.,* 2012), their developmental stages (Bidartondo & Read, 2008; Těšitelová *et al.,* 2015; Waud *et al.,* 2017), ecological conditions (Bidartondo *et al.,* 2004; Jacquemyn *et al.,* 2016; Duffy *et al.,* 2019) and also trophic modes (Motomura *et al.,* 2010; Ogura‐Tsujita *et al.,* 2012). Autotrophic orchids generally associate with rhizoctonia fungi, while partially and fully mycoheterotrophic orchids mostly associate with ECM and wood‐ and litter‐decaying fungi, suggesting that transitions in trophic modes are probably linked to shifts in fungal lifestyles in Orchidaceae (Jacquemyn & Merckx, 2019). Detailed studies on Neottieae (Selosse *et al.,* 2004; Selosse & Roy, 2009; Yagame *et al.,* 2016) and *Cymbidium* (Ogura‐Tsujita *et al.,* 2012) have proposed an evolutionary correlation between fungal lifestyle and trophic mode in these taxa. However, this evolutionary correlation remains to be tested in a comprehensive family‐wide phylogenetic framework.

In the past two decades, the ecophysiology of orchids and the identity of their fungal partners have been studied extensively, fueled by the development of stable isotope measurements and novel DNA sequencing methods (Gebauer & Meyer, 2003; Bidartondo *et al.,* 2004; Martos *et al.,* 2009; Jacquemyn *et al.,* 2017b; Schiebold *et al.,* 2017, 2018; Schweiger *et al.,* 2019). In this study, we compiled a family‐wide dataset of mycorrhizal interactions and orchid trophic modes, and employed a phylogenetic framework to test the hypothesis that shifts in trophic mode in the orchid family are correlated with switches in fungal communities. Specifically, we aimed to answer the following questions: what is the evolutionary history of fungal associations and trophic modes within the family is the evolution towards mycoheterotrophy correlated with switches in fungal communities and what is the most common evolutionary scenario towards mycoheterotrophy in Orchidaceae in the context of fungal partner switches?

## Materials and Methods

### Orchid mycorrhiza, fungal lifestyle, and trophic mode

To compile a family‐wide dataset of orchid fungal associations, we searched for molecular data using the keywords ‘orchid mycorrhizal fungi’ in Mendeley Reference Management Desktop (before August 2019) and only retained articles that contained fungal nuclear ribosomal internal transcribed spacer (ITS) accessions generated with either Sanger or high‐throughput sequencing (HTS) techniques. By manually checking and filtering the obtained articles, *c*. 250 were kept as original references for the orchid mycorrhiza dataset. The final dataset contained information on 750 orchid species covering nearly all major clades of orchids (20 tribes and 39 subtribes belonging to five subfamilies) (see Supporting Information Table S1). In addition, we searched for isotope data of the orchid species included in our mycorrhizal dataset. For each orchid species, all available DNA accessions of the markers’ ITS, *matK*, *rbcL* and *trnL‐F*, were downloaded from the NCBI GenBank database.

Taxonomic information was assigned to each fungal sequence using Usearch v.11 (Edgar, 2010). We further categorized the sequences at the family level because half of all sequences could not be assigned to a lower taxonomic level (genus or species) by blasting against the UNITE local database (Abarenkov *et al.,* 2010). Detailed procedures of fungal operational taxonomic unit (OTU) clustering and taxonomic assignment are described in Notes S1. After removing fungal families associated with only a single orchid species, we assigned the remaining fungal families in Basidiomycota and Ascomycota to different lifestyles based on the information provided in the original publications (Dearnaley *et al.,* 2012; Tedersoo & Brundrett, 2017; Põlme *et al.,* 2020) supplemented by funguild (Nguyen *et al.,* 2016) using default parameters. We categorized the rhizoctonia families (Tulasnellaceae, Ceratobasidiaceae, and Serendipitaceae – Sebacinales ‘group B’) as ‘rhizoctonia‐like’ fungi (RHI). Families mainly comprising ECM fungi (Sebacinaceae – Sebacinales ‘group A’, Thelephoraceae, Russulaceae, and others) were classified as ECM. Families mainly containing saprotrophic fungi (e.g. Mycenaceae, Psathyrellaceae) were classified as saprotrophic (SAP). Fungal families having both ECM and SAP fungi were classified as the ECM/SAP lifestyle. Because a large number of fungal families contain members that are pathogens, endophytes, or belong to unknown ecological guilds, we adopted a conservative approach in all further analyses by restricting the number of fungal families to the 17 families that are known to contain putative orchid mycorrhizal fungi (Table 12.1 in Dearnaley *et al.,* 2012).

Based on morphological descriptions and stable isotope signatures (^13^C and ^15^N), we assigned orchid species to three trophic modes: autotrophy (AU), partial mycoheterotrophy (PMH), and full mycoheterotrophy (MH). Due to the inconsistency in ^13^C and ^15^N signatures of several orchids, either a strict or more relaxed definition of PMH was adopted. A detailed description of the criteria to assign an orchid to one of the trophic modes can be found in Notes S1.

### Orchid phylogeny reconstruction and divergence time estimation

To reconstruct a time‐calibrated phylogeny of Orchidaceae, we performed a relaxed molecular clock analysis with Beast v.2.5 (Bouckaert *et al.,* 2019) using backbone trees from Chase *et al.,* (2015), Chomicki *et al.,* (2015) and Givnish *et al.,* (2015). Sequence alignment, phylogenetic reconstruction, and time calibration are described in detail in the Notes S1.

### Phylogenetic signal

Pagel’s lambda (λ) (Pagel, 1999) was used to investigate whether the distribution of the character states of trophic mode and fungal lifestyle showed some degree of phylogenetic signal on the orchid phylogeny. The λ value was estimated using ‘fitDiscrete’ function in the R package geiger (Pennell *et al.,* 2014).

### Ancestral state estimations

Ancestral state reconstruction was used to trace the evolutionary history of trophic modes and symbiotic associations represented by fungal lifestyles over the reconstructed orchid phylogeny. Ancestral state reconstructions were performed with the ‘make.simmap’ function in the R package phytools (Revell, 2012) using stochastic character mapping (Bollback, 2006). An initial model with independent rates of state transitions (‘All Rates Different’, ARD) was applied among symbiotic associations and trophic modes, respectively. Because MH is associated with rampant plastid gene loss (Graham *et al.,* 2017) and is thus likely irreversible (Merckx, 2013), we specified the transition rates from MH to PMH and AU as zero in the state transition rate matrix (Q matrix). The ancestral states of trophic mode were inferred based on both a relaxed and a strict definition of PMH. In addition, analyses of ancestral state reconstruction were also performed using ‘MultiState’ Markov chain Monte Carlo (MCMC) analysis implemented in bayestraits v.3, adopting the same model for each discrete trait as with stochastic character mapping. We ran each of our analyses for 1010 000 iterations with the first 10 000 generations as burn‐in. Reversible‐jump MCMC analyses (Green, 1995; Pagel & Meade, 2006) were applied to reduce model complexity and over‐parameterization.

### Hypothesis tests

Discrete Independent and Dependent models in bayestraits detected correlated trait evolution between symbiotic association and trophic mode (Notes S1). However, with this approach, the path of trait evolution was not yet evident. Therefore, we combined the two traits into a coupled multistate character and performed explicit hypothesis tests to verify plausible evolutionary scenarios towards MH. By setting constraints on the Q matrix, we tested which state was best supported to be the intermediate state that enabled the evolution of mycoheterotrophy (Notes S1). To compare models, we estimated the marginal likelihood of the free model where no constraints were set to the Q matrix. One constrained model disallowing specific transitions between states in the Q matrix that best fitted the data will result in the marginal likelihood that differs most significantly from the less constrained free model. We ran each ‘MultiState’ analysis in triplicate for 10^6^ generations and calculated the average marginal likelihood using a stepping stone sampler. Finally, we compared the constrained models with the free model by Bayesian information criterion (BIC), and the model with the lowest BIC value was selected.

## Results

### Mycorrhizal interactions in Orchidaceae

**Fig. 1:**
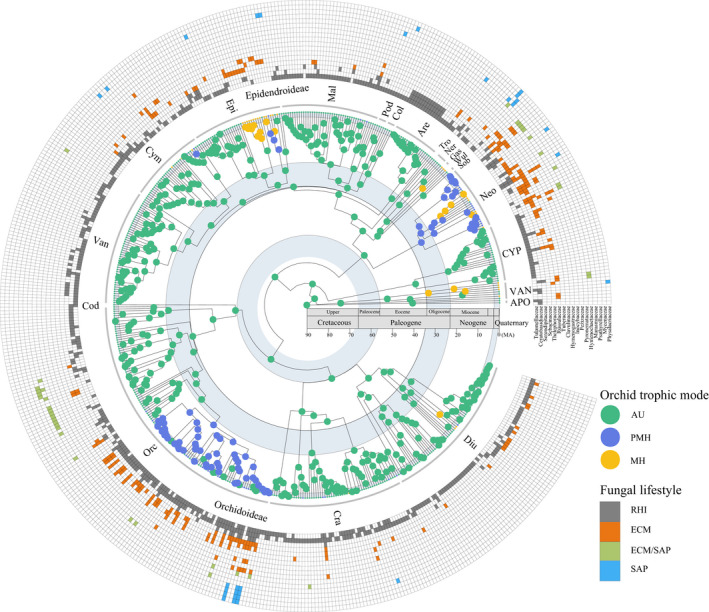
Orchid phylogeny, ancestral state reconstruction of trophic mode and symbiotic association. The orchid chronogram is annotated with subfamilies and tribes represented by three‐letter initials (see the full name in Supporting Information Table S2); the highest posterior probability state of trophic modes is mapped on all internal nodes of the tree based on ancestral state reconstruction (Fig. S5). AU, autotrophy; PMH, partial mycoheterotrophy; MH, full mycoheterotrophy. The geological time scales are visualized by circles from upper Cretaceous (*c*. 90–66 million yr ago (Ma)), via Paleocene (*c*. 66–23 Ma), and Neogene (*c*. 23–2.58 Ma), to Quaternary (from 2.58 Ma to the present). The outside matrix represents the presence or absence of 17 fungal families containing putative orchid mycorrhizal fungi (Dearnaley *et al.,* 2012). Fungal families are ranked by lifestyles and the number of orchid species they associate with. The lifestyles (‘rhizoctonia‐like’ fungi (RHI), ectomycorrhizal (ECM), ECM/saprotrophic (ECM/SAP), and SAP) of each fungal family are visualized using different colors. Visualization was generated using the R package ggtree (Yu, 2020).

Adopting a strict definition of PMH, a total of 455 AU, 27 PMH, and 37 MH orchid species were used for phylogenetic reconstruction (Table S2). Under a relaxed definition of PMH, there were 414 AU, 69 PMH species, and 37 MH species (Table S2). After removing fungal families that associated with a single orchid species, a total of 68 fungal families in Basidiomycota and Ascomycota were identified based on ITS sequences generated from both Sanger and HTS techniques (Fig. S1). Autotrophic orchids (455 species) were associated with the highest diversity of fungal taxa (66 fungal families), and shared 67% and 50% of their fungal families with PMH and MH orchids, respectively (Fig. S2). The number of fungal families detected in our dataset showed small differences between Sanger sequencing and HTS techniques (see details in Notes S1; Fig. S3). Seventeen of all detected fungal families that have previously been identified as putative orchid mycorrhiza fungi (Dearnaley *et al.,* 2012) were mapped on the orchid phylogeny (Figs 1, S4), including three rhizoctonia families (Tulasnellaceae, Ceratobasidiaceae and Serendipitaceae), seven families mainly comprising ECM fungi (Sebacinaceae, Thelephoraceae, Russulaceae, Tuberaceae, Clavulinaceae, Hymenogastraceae, Inocybaceae), and seven families containing SAP and/or ECM fungi (Pezizaceae, Pyronemataceae, Hymenochaetaceae, Marasmiaceae, Psathyrellaceae, Mycenaceae, Physalacriaceae). The three rhizoctonia families were present in the roots of 454 (87.5%) of 519 orchid species, followed by major ECM families (e.g. Sebacinaceae, Thelephoraceae, and Russulaceae) (Figs 1, S4).

### Ancestral states of trophic mode and symbiotic association

**Fig. 2:**
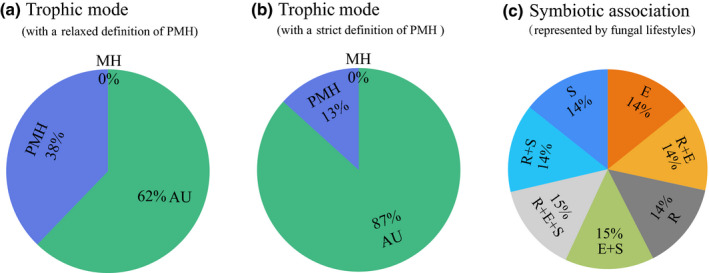
Ancestral state reconstruction of trophic mode and symbiotic association using bayestraits ‘MultiState’ analysis. (a) The posterior probability of trophic modes of the common ancestor of orchids under a relaxed definition of partial mycoheterotrophy (Supporting Information Notes S1). AU, autotrophy; PMH, partial mycoheterotrophy; MH, full mycoheterotrophy. (b) The posterior probability of trophic modes of the common ancestor of orchids under a strict definition of PMH (Notes S1). (c) The posterior probability of symbiotic associations of the common ancestor of orchids. Symbiotic associations are represented by the lifestyles of fungal families that one orchid associates with. R, ‘rhizoctonias’; E, ectomycorrhizal fungi; S, saprotrophic fungi.

Both stochastic character mapping and ‘MultiState’ MCMC analysis inferred that the common ancestor of orchids was most likely autotrophic at adult stage (Figs 2a,b, S5, S6). From the ancestral state of AU, there have been 42 or 28 transitions to PMH, and 17 or 18 shifts to MH by using the relaxed and strict definition of PMH, respectively (Figs S5, S6). Few transitions were detected directly from AU to MH using both definitions of PMH (Figs S5, S6). Shifts to mycoheterotrophy mainly occurred in the subfamilies Vanilloideae (Vanilleae), Orchidoideae (Orchideae, Cranichideae, Diurideae), and Epidendroideae (Neottieae, Gastrodieae, Wullschlaegelieae, Epidendreae, Cymbidieae) (Figs S5, S6). By tracing transitions in trophic modes through geological time, we showed a general trend of transitions from AU towards PMH and MH, and transitions to MH were predated by transitions to PMH (Fig. S7a). Full mycoheterotrophy appeared only recently in the evolutionary history of Orchidaceae at the start of the Oligocene (*c*. 35 million years ago; Fig. S7a).

**Fig. 3:**
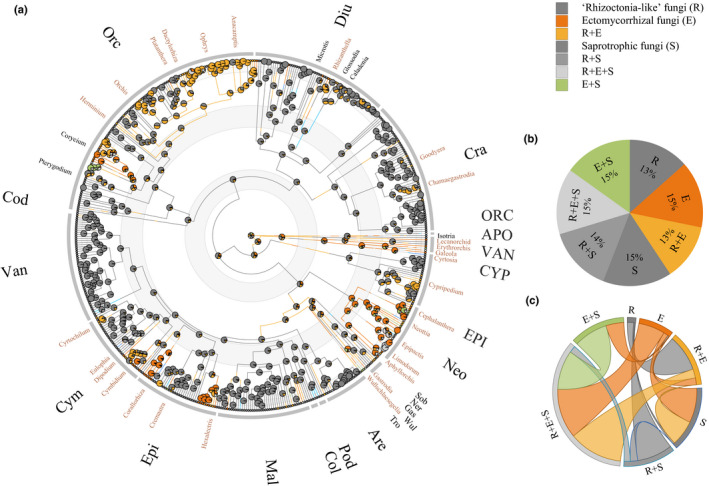
Ancestral state reconstruction of symbiotic association in Orchidaceae. (a) Likelihood pie reconstruction of each symbiotic association for all nodes. The symbiotic association used for ancestral state estimation is based on the lifestyles of 17 fungal families containing putative orchid mycorrhizal fungi (Dearnaley *et al.,* 2012). The orchid chronogram is annotated with subfamilies and tribes represented by three‐letter initials (see the full name in Supporting Information Table S2), and genera where shifts to ectomycorrhizal or saprotrophic fungi occur. The genera comprising mycoheterotrophic species are colored in brown. The geological time scales are visualized by circles from upper Cretaceous (*c*. 90–66 million yr ago (Ma)), via Paleocene (*c*. 66–23 Ma), and Neogene (*c*. 23–2.58 Ma), to Quaternary (from 2.58 Ma to the present). (b) The likelihood of each symbiotic association for the root. R, ‘rhizoctonias’, E, ectomycorrhizal fungi, S, saprotrophic fungi. (c) The transitions between symbiotic associations. The band size for each symbiotic association represents the proportion of transitions among all transitions; the width of the ribbons represents the proportion of transitions starting from that state.

Ancestral state reconstruction using both methods of stochastic character mapping and ‘MultiState’ MCMC analysis inferred that each symbiotic association has a comparatively equal probability of the ancestral state (Figs 2c, 3), indicating that the ancestor of orchids was probably associated with a broad range of fungi. Most transitions started from the combined state R + E + S to a variety of symbiotic associations, mainly including ECM and SAP fungi (Fig. 3c). The majority of transitions to ECM and wood‐ or litter‐decaying SAP fungi involved 34 orchid genera belonging to nine tribes (Fig. 3), of which six genera do not contain PMH or MH species (Table S2). By trancing transitions in symbiotic associations through geological time, we inferred a general trend of shifts from a fungal community comprising diverse fungal lifestyles in the ancestor of orchids towards more specialized fungal lifestyles among extant lineages of Orchidaceae (Fig. S7b). A large proportion of evolutionary shifts towards an exclusive association with rhizoctonia‐like fungi was observed, whereas only a small proportion of transitions towards ECM and wood‐ or litter‐decaying SAP fungi was detected (Fig. S7b).

### Phylogenetic signals and trait correlations

We detected a robust phylogenetic signal for both trophic mode and fungal lifestyle (Table S3). A strong correlation between trophic mode and symbiotic association was inferred by both the Discrete Independent and Dependent models, as supported by Bayes factor scores (Table S4). Furthermore, our ‘MultiState’ analyses of trophic mode and symbiotic association showed that the fourth model is favored with the lowest BIC compared with the other three models, which sets constraints on the forward transition from autotrophic states (states 1, 2 and 3) to mycoheterotrophic states (states 4 and 5) (Table 1). This model assumes that a forward transition from AU to MH is associated with an obligate intermediate transition of symbiotic association – a combination of rhizoctonias and ECM or SAP fungi (Notes S1). By setting further constraints on the reverse direction of transitions, our results showed that the sixth model best fitted our data (Table 1). This model assumes that the reverse transition from the MH to the AU state only occurs from the intermediate symbiotic association with a combination of rhizoctonias and ECM or SAP fungi (Notes S1).

**Table 1:** Model tests of possible evolutionary scenarios of the coupled character combining trophic mode and symbiotic association using ‘MultiState’ analyses implemented in bayestraits.

Models	No. of parameters	Log_e_ *L*	BIC
The free model	20	−487.99	1101.01
Model 1	16	−499.07	1098.17
Model 2	16	−486.45	1072.93
Model 3	16	−484.67	1069.37
Model 4	15	−485.3	1064.37
Model 5	12	−481.95	1038.92
Model 6	11	−479.63	1028.03

BIC, Bayesian information criterion.

Trophic mode (autotrophy and full mycoheterotrophy) and symbiotic association represented by fungal lifestyle (‘rhizoctonias’ (R), ectomycorrhizal fungi (E) and saprotrophic fungi (S)) in our dataset were converted into a five‐state character: (1) AU‐R; (2) AU‐ES; (3) AU‐RES; (4) MH‐RES; and (5) MH‐ES. We developed plausible models to test different evolutionary paths towards full mycoheterotrophy contributed by symbiotic shifts (see the diagram of models in Supporting Information Notes S1). The BIC is used to compare models, and the model with the lowest BIC values is selected.

## Discussion

### Evolutionary shifts in symbiotic associations

Our results suggest that the common ancestor of orchids probably did not associate with fungi of a single lifestyle, but rather with a fungal community of multiple lifestyles (Figs 2c, 3). Although rhizoctonias were most likely part of this community, as previously suggested by Yukawa *et al*. (2009), our analyses indicate the possibility of a much wider partner breadth in the ancestors of orchids (Figs 2c, 3). Our results further show that the largest proportion of evolutionary shifts in symbiotic associations occurred from the combined state of rhizoctonias, ECM, and SAP fungi (R + E + S) towards more specialized associations (Fig. 3c). Similarly, a dual symbiotic association was also recently inferred as a prerequisite for shifts between major mycorrhizas among land plants (Werner *et al.,* 2018). These studies collaboratively support the overall hypothesis that the evolution of symbiotic shifts follows a stepwise process, in which fungal partners in a later stage of a mycorrhizal association have been latently present in the fungal community of the ancestor (Selosse *et al.,* 2010; van der Heijden *et al.,* 2015; Jacquemyn & Merckx, 2019; Suetsugu & Matsubayashi, 2021).

Support for this so‐called ‘waiting room’ hypothesis (Selosse *et al.,* 2010, 2018; van der Heijden *et al.,* 2015) has been mounting. For instance, several ECM fungi colonizing orchid roots are also endophytes in surrounding plants or even in orchid tissues, such as Sebacinaceae (Selosse *et al.,* 2009; Oliveira *et al.,* 2014; Weiß *et al.,* 2016) and Tuberaceae (Gryndler *et al.,* 2014; Schneider‐Maunoury *et al.,* 2018). Likewise, some groups of free‐living SAP fungi have been suggested to transit to a mycorrhizal association with orchid species through an endophytic state, such as Mycenaceae (Martos *et al.,* 2009; Ogura‐Tsujita *et al.,* 2009) and Psathyrellaceae (Yamato *et al.,* 2005; Yagame *et al.,* 2007; Ogura‐Tsujita & Yukawa, 2008). These findings suggest that the ability to associate with non‐mycorrhizal fungi is probably a predisposition for tight mycorrhizal associations in Orchidaceae (e.g. Shubin *et al.,* 2009). However, due to the lack of microscope observations or germination tests, we cannot be sure that all fungal taxa recorded in this study are truly mycorrhizal, and thus some might reside in orchid roots as endophytes. Considering that a large number of fungal associates of orchids was recorded in this study (Figs S1, S2), previous studies, especially those that have restrictions in primer sets and sequencing methods, may have overlooked a wide range of fungal associates that reside in orchid roots (Selosse *et al.,* 2010, 2018).

### Evolutionary shifts in trophic modes

Our analyses further show that from the ancestral autotrophic state (Figs 1, 2a,b), shifts from AU or PMH to MH have occurred at least 17 times across the orchid family (Figs S5, S6), accounting for a large fraction of the estimated total of 40 of these shifts across land plants (Merckx & Freudenstein, 2010; Merckx, 2013; Jacquemyn & Merckx, 2019). The occurrence of mycoheterotrophy in orchids exhibits phylogenetic conservatism and is mainly confined to nine tribes in the subfamilies Vanilloideae, Orchidoideae, and Epidendroideae (Figs S5, S6). In addition, a recent study has shown that in the early divergent subfamily Apostasioideae a photosynthetic orchid species is potentially mycoheterotrophic due to its enriched ^13^C and ^15^N signatures (Suetsugu & Matsubayashi, 2021). As the ^13^C and ^15^N signatures of only few species have been investigated, and because these isotopes cannot detect low degrees of PMH in orchids, especially for those associated with rhizoctonias (Gebauer *et al.,* 2016; Schiebold *et al.,* 2018; Schweiger *et al.,* 2019), the number of shifts to mycoheterotrophy may have been underestimated here.

Transitions to MH occurred dominantly through an intermediate stage of PMH (Figs S5, 6), which is in line with previous phylogenetic studies on narrower phylogenetic scales (Selosse & Roy, 2009; Motomura *et al.,* 2010; Ogura‐Tsujita *et al.,* 2012; Yagame *et al.,* 2016; Jacquemyn & Merckx, 2019). Furthermore, stable isotope studies have confirmed that ^13^C and ^15^N signatures of PMH orchids are on a dynamic continuum between AU and MH (Jacquemyn *et al.,* 2017b; Schiebold *et al.,* 2018; May *et al.,* 2020). These findings corroborate the hypothesis that transitions to MH are accomplished by a gradual increase in the level of mycoheterotrophy, rather than by abrupt shifts of AU to MH. This gradual transition has also been suggested in the genus *Pyrola* in the family Ericaceae, where a similar full range of trophic modes has been observed (Tedersoo *et al.,* 2007; Zimmer *et al.,* 2007; Hynson *et al.,* 2009).

### Correlation between symbiotic switches and trophic mode shifts

Ancestral state reconstructions show that symbiotic switches often co‐occur with shifts in trophic modes (Figs 1, 3, S4–S6). Discrete Independent and Dependent models further revealed a strong correlation between transitions in trophic modes and fungal partners across the orchid phylogeny (Table S4). Such correlation has been suggested based on observations in genus‐level analyses (Bidartondo *et al.,* 2004; Selosse *et al.,* 2004; Motomura *et al.,* 2010; Ogura‐Tsujita *et al.,* 2012; Jacquemyn *et al.,* 2016, 2017b; Yagame *et al.,* 2016). Fully mycoheterotrophic orchids primarily associate with either ECM or wood and litter‐decaying SAP fungi, while rhizoctonias dominate the associations of their autotrophic relatives (Motomura *et al.,* 2010; Ogura‐Tsujita *et al.,* 2012; Yagame *et al.,* 2016), suggesting that full or partial loss of photosynthesis selects for different mycorrhizal communities. Therefore, the evolutionary transition from AU over PMH to MH was accompanied by a shift in fungal partners from rhizoctonias to ECM or SAP fungi in the orchid family (Figs 1, 3; Table 1). The latter fungal groups are often detected alongside rhizoctonia‐like fungi in the roots of autotrophic orchids, and here we infer that the association with a broad range of fungal partners was the ancestral state in orchids (Fig. 3). The symbiotic shift to ECM or wood‐ and litter‐decaying saprotrophic fungi is therefore likely an essential predisposition for the evolution of mycoheterotrophy (Table 1). The trophic mode shift to mycoheterotrophy seems thus to go in parallel with increased importance of ECM and SAP fungi while gradually discarding the rhizoctonias that were associated with the autotrophic ancestors.

In arbuscular and ECM systems, several experimental studies have shown that plants can reward greater nutrient‐providing symbionts with increased carbon supplies, and thus ‘choose’ optimal fungal partners from the local environment (Bever *et al.,* 2009; Kiers *et al.,* 2011; Bogar *et al.,* 2019). An increased level of mycoheterotrophy requires an increased dependence on fungal carbon, ultimately replacing photosynthesis in MH plants. Because carbon is the primary resource that non‐photosynthetic mycoheterotrophic plants receive from their mycorrhizal interactions, Taylor & Bruns (1997) hypothesized that the driving force for orchids to change their fungal partners is to obtain a more stable and higher amount of carbon and/or nitrogen. It has been suggested that ECM fungi, which are tightly associated with forest trees, or SAP fungi living on dead wood or decaying leaves are more beneficial partners for mycoheterotrophic orchids in deeply shaded forest habitats than rhizoctonias, which may have limited SAP capabilities (Roberts, 1999). Recent genomic studies have shown that multiple lineages of ECM fungi exhibit a reduced capability to acquire C from soil organic matter and plant cell walls, which is elucidated by rampant loss of genes encoding lignocellulose‐degrading enzymes present in their SAP ancestors (Miyauchi *et al.,* 2020). Interestingly, ECM fungi have evolved novel and species‐specific genes, which may contribute to their tight symbiotic associations with forest trees (Kohler *et al.,* 2015; Hess *et al.,* 2018; Miyauchi *et al.,* 2020), and in this way provide more stable nutrient supplies to nearby orchids species in the tripartite network (Merckx, 2013).

## Author contributions

VSFTM initiated and supervised the project. DW compiled the data and performed the analyses with input from HJ, SIFG, and RAV All authors contributed to writing of the manuscript.

## Supporting information


**Fig. S1** The number of orchid species associated with each fungal family.
**Fig. S2** Fungal family composition among orchid species with different trophic modes.
**Fig. S3** A comparison between Sanger sequencing and high‐throughput sequencing (HTS) techniques.
**Fig. S4** Orchid phylogeny, ancestral states of trophic mode, and symbiotic association.
**Fig. S5** Ancestral state reconstruction of the trophic mode under a relaxed definition of partial mycoheterotrophy (PMH) using stochastic character mapping.
**Fig. S6** Ancestral state reconstruction of the trophic mode under a strict definition of partial mycoheterotrophy (PMH) using stochastic character mapping.
**Fig. S7** Changes of trophic mode and symbiotic association through time.
**Notes S1** Additional methodological details and results.

**Table S1** Orchid mycorrhiza dataset.
**Table S2** The list of orchid species for phylogenetic reconstruction and trait analyses.
**Table S3** Phylogenetic signals of symbiotic association and trophic mode using Pagel’s lambda.
**Table S4** Phylogenetic correlations between orchid trophic mode and symbiotic association using the Discrete Independent and Dependent models implemented in bayestraits.
Please note: Wiley Blackwell are not responsible for the content or functionality of any Supporting Information supplied by the authors. Any queries (other than missing material) should be directed to the *New Phytologist* Central Office.
