# Supplementary material for: Symbiont switching and trophic mode shifts in Orchidaceae
Source: New Phytol. 2021 Jun 1;231(2):791–800. doi: 10.1111/nph.17414 (PMC8252101; doi:10.1111/nph.17414)
Supplement: Supplementary file 1 — Fig. S1 The number of orchid species associated with each fungal family. Fig. S2 Fungal family composition among orchid species with different trophic modes. Fig. S3 A comparison between Sanger sequencing and high‐throughput sequencing (HTS) techniques. Fig. S4 Orchid phylogeny, ancestral states of trophic mode, and symbiotic association. Fig. S5 Ancestral state reconstruction of the trophic mode under a relaxed definition of partial mycoheterotrophy (PMH) using stochastic character mapping. Fig. S6 Ancestral state reconstruction of the trophic mode under a strict definition of partial mycoheterotrophy (PMH) using stochastic character mapping. Fig. S7 Changes of trophic mode and symbiotic association through time. Notes S1 Additional methodological details and results. [file NPH-231-791-s002.pdf]

New Phytologist

**_New Phytologist_ Supporting Information**

Article title: Symbiont Switching and Trophic Mode Shifts in Orchidaceae
Authors: Deyi Wang[1,2*], Hans Jacquemyn[3], Sofia I.F. Gomes[4], Rutger A. Vos[1,2], Vincent S.F.T. Merckx[1,5]
Article acceptance date: 09 April 2021

The following Supporting Information is available for this article:

**Fig. S1** The number of orchid species per fungal family associated with.

**Fig. S2** Fungal family composition among orchid species with different trophic modes.

**Fig. S3** A comparison between Sanger sequencing and high-throughput sequencing (HTS) techniques.

**Fig. S4** Orchid phylogeny, ancestral states of trophic mode, and symbiotic association.

**Fig. S5** Ancestral state reconstruction of the trophic mode under a relaxed definition of partial mycoheterotrophy (PMH) using stochastic character mapping.

**Fig. S6** Ancestral state reconstruction of the trophic mode under a strict definition of partial mycoheterotrophy (PMH) using stochastic character mapping.

**Fig. S7** Changes of trophic mode and symbiotic association through time.

**Table S1** Orchid mycorrhiza dataset.

**Table S2** The list of orchid species for phylogenetic reconstruction and trait analyses.

**Table S3** Phylogenetic signals of symbiotic association and trophic mode using Pagel's lambda.

**Table S4** Phylogenetic correlations between orchid trophic mode and symbiotic association using the Discrete Independent and Dependent models implemented in BayesTraits.

**Notes S1** Additional methodological details and results.

**Fig. S1 The number of orchid species per fungal family associated with.** The sixty-eight fungal families in Basidiomycota and Ascomycota excluding singletons are ranked by the number of orchid species that they associate with.

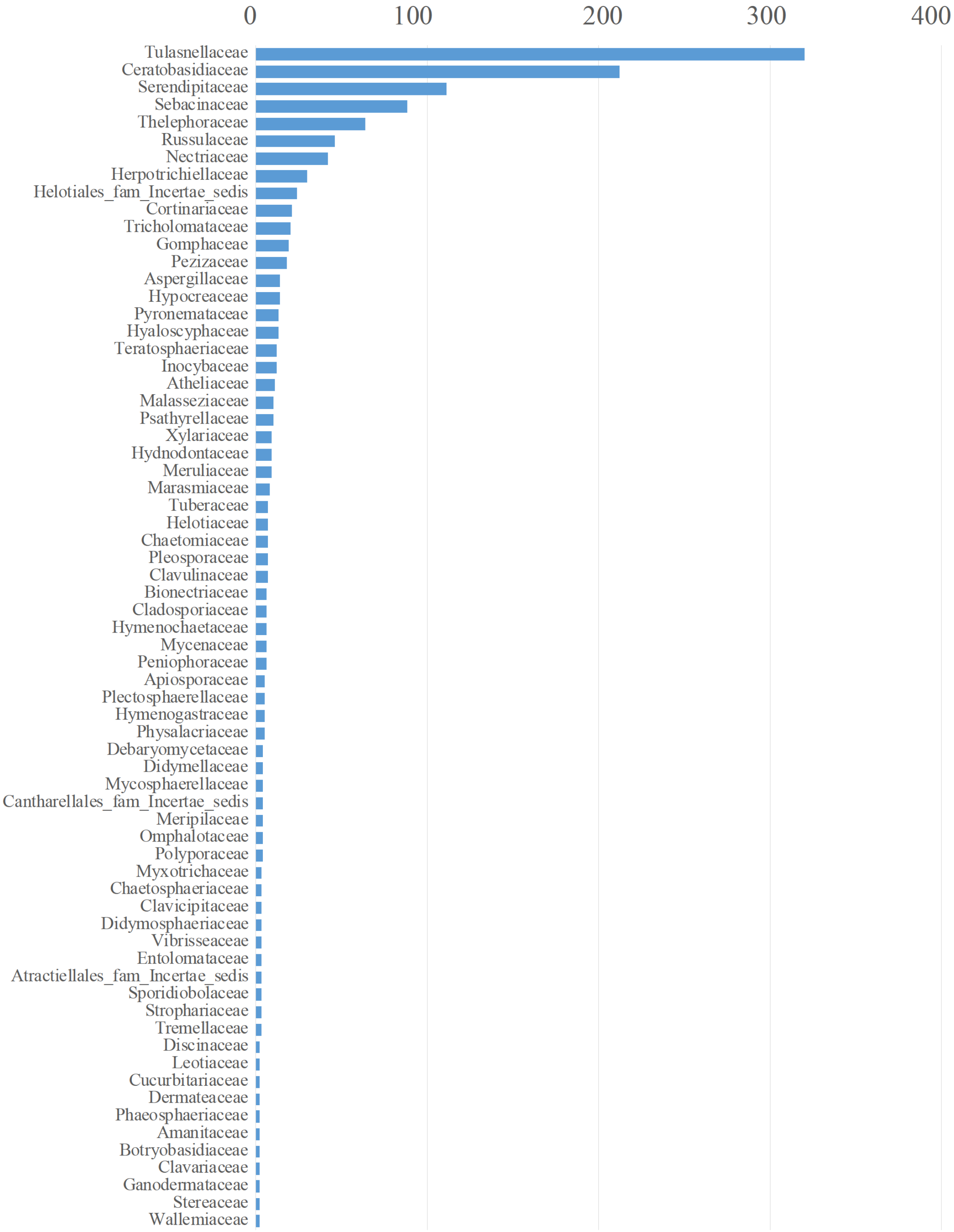

**Fig. S2 Fungal family composition among orchid species with different trophic modes.** AU: autotrophy; PMH: partial mycoheterotrophy (a relaxed definition; see Supporting information Notes S1); MH: full mycoheterotrophy.

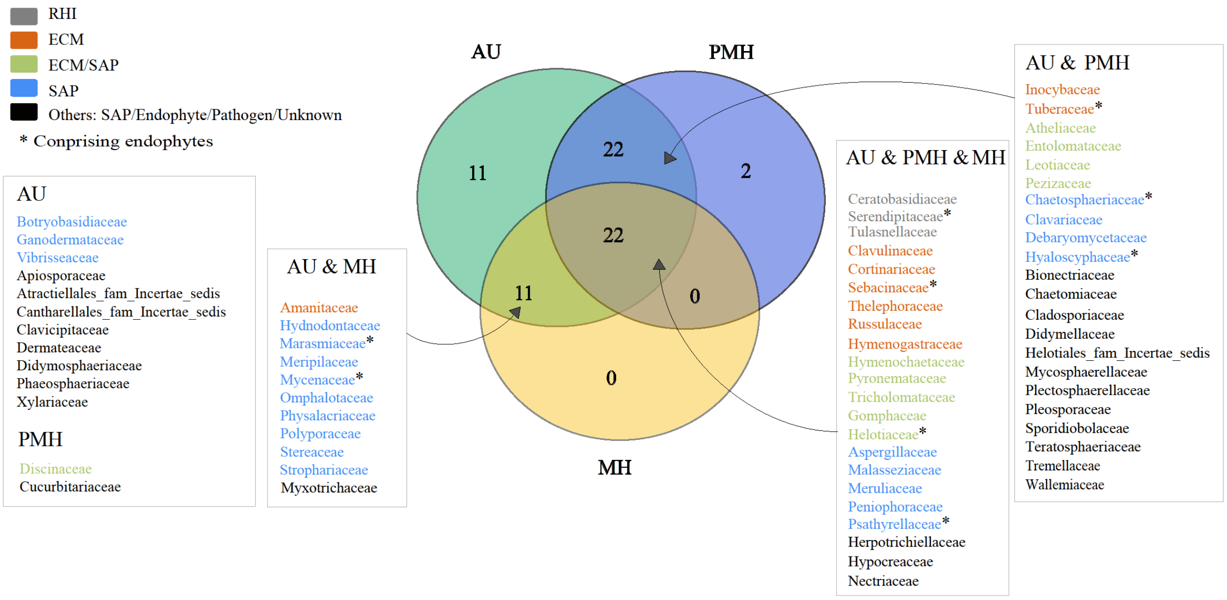

**Fig. S3 A comparison between Sanger sequencing and high-throughput sequencing (HTS) techniques.** (a) The number of fungal families per orchid species associated with regarding different sequencing techniques based on the fungal associates of 519 orchid species. (b) The number of fungal families per orchid species associated with based on the fungal associates of eleven orchid species generated from both sequencing techniques. (c) The average number of studies on the eleven orchid species that adopted both sequencing techniques.

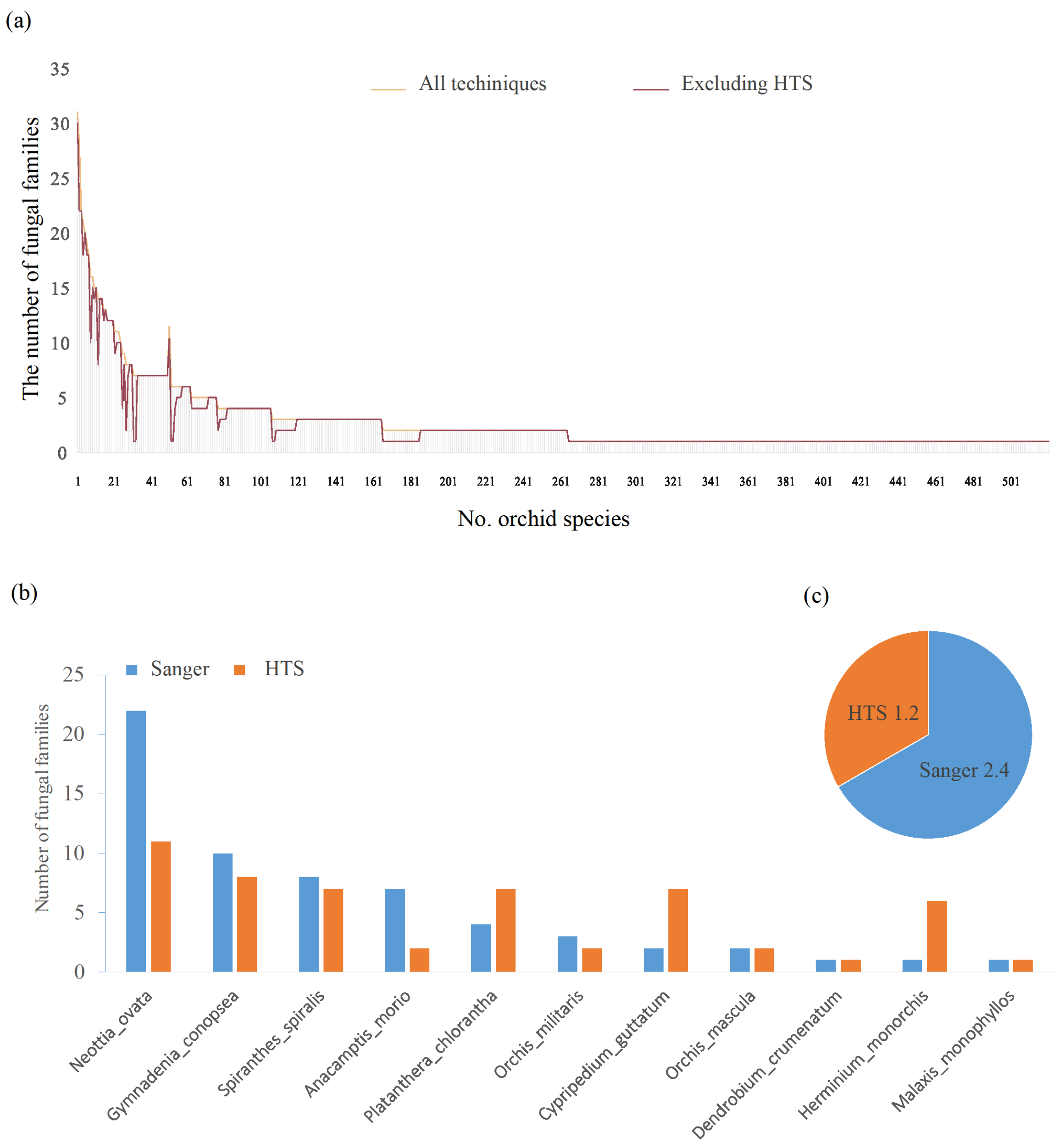

**Fig. S4 Orchid phylogeny, ancestral states of trophic mode, and symbiotic association.** The orchid phylogeny is annotated with subfamilies, tribes, and species; the pie chart of the posterior probability state of trophic modes under a relaxed definition of partial mycoheterotrophy (Supporting information Notes S1) is mapped on all internal nodes of the tree. The geological time scales are visualized by circles from upper Cretaceous (*c.* 90 – 66 MA), via Paleocene (*c.* 66 – 23 MA), and Neogene (*c.* 23 – 2.58 MA), to Quaternary (from 2.58 MA to the present). The matrix on the right matches the tips of the orchid phylogeny, and represents the presence or absence of seventeen fungal families containing putative orchid mycorrhizal fungi (Dearnaley *et al.*, 2012). Fungal families are ranked by the number of orchid species that they associate with. Trophic modes (AU, PMH, and MH) and symbiotic associations represented by fungal lifestyles (RHI, ECM, ECM/SAP, and SAP) are visualized using different colors. Visualization generated using "ggtree" R package (Yu 2020).

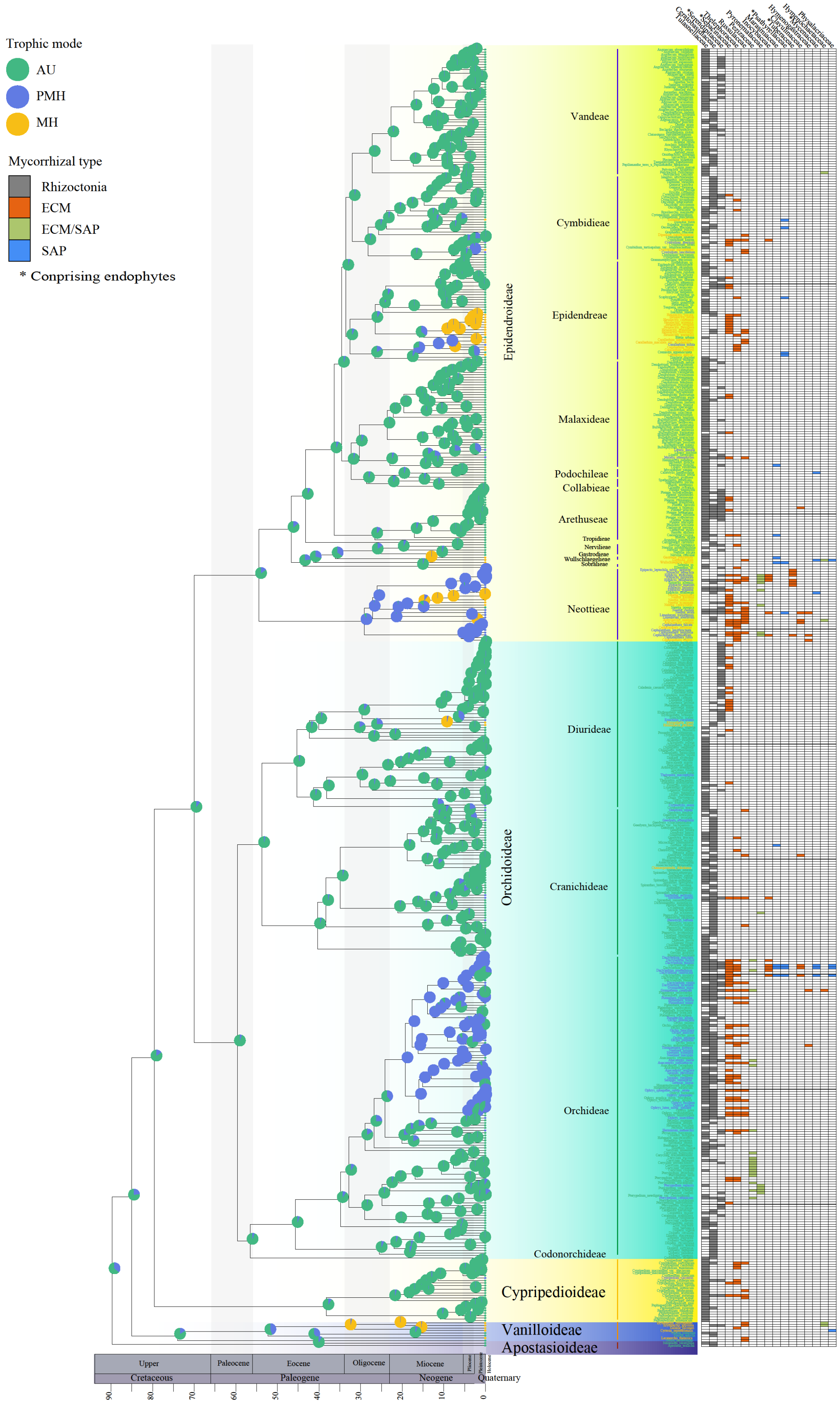

Trophic mode
AU
PMH
MH
Mycorrhizal type
Rhizoctonia
ECM
ECM/SAP
SAP
* Conprising endophytes
Vandeae
Cymbidieae
Epidendreae
Malaxideae
Podochileae
Collabieae
Arethuseae
Tropidieae
Nervilieae
Gastrodieae
Wullschlaegelieae
Sobralieae
Neottieae
Epidendroideae
Diurideae
Cranichideae
Orchideae
Codonorchideae
Orchidoideae
Cypripedioideae
Vanilloideae
Apostasioideae
Upper
Cretaceous
Paleocene
Eocene
Paleogene
Oligocene
Miocene
Neogene
Pliocene
Pleistocene
Holocene
Quaternary
90
80
70
60
50
40
30
20
10
0

**Fig. S5 Ancestral state reconstruction of the trophic mode under a relaxed definition of partial mycoheterotrophy (PMH) using stochastic character mapping.** Using a relaxed definition of PMH (Supporting information Notes S1), orchid species are enriched in only $^{13}C$ or $^{15}N$ compared with surrounding autotrophic vegetation. The number of transitions between trophic modes is visualized at the bottom right of the figure. The geological time scales are visualized by circles from upper Cretaceous (*c.* 90 – 66 MA), via Paleocene (*c.* 66 – 23 MA), and Neogene (*c.* 23 – 2.58 MA), to Quaternary (from 2.58 MA to the present).

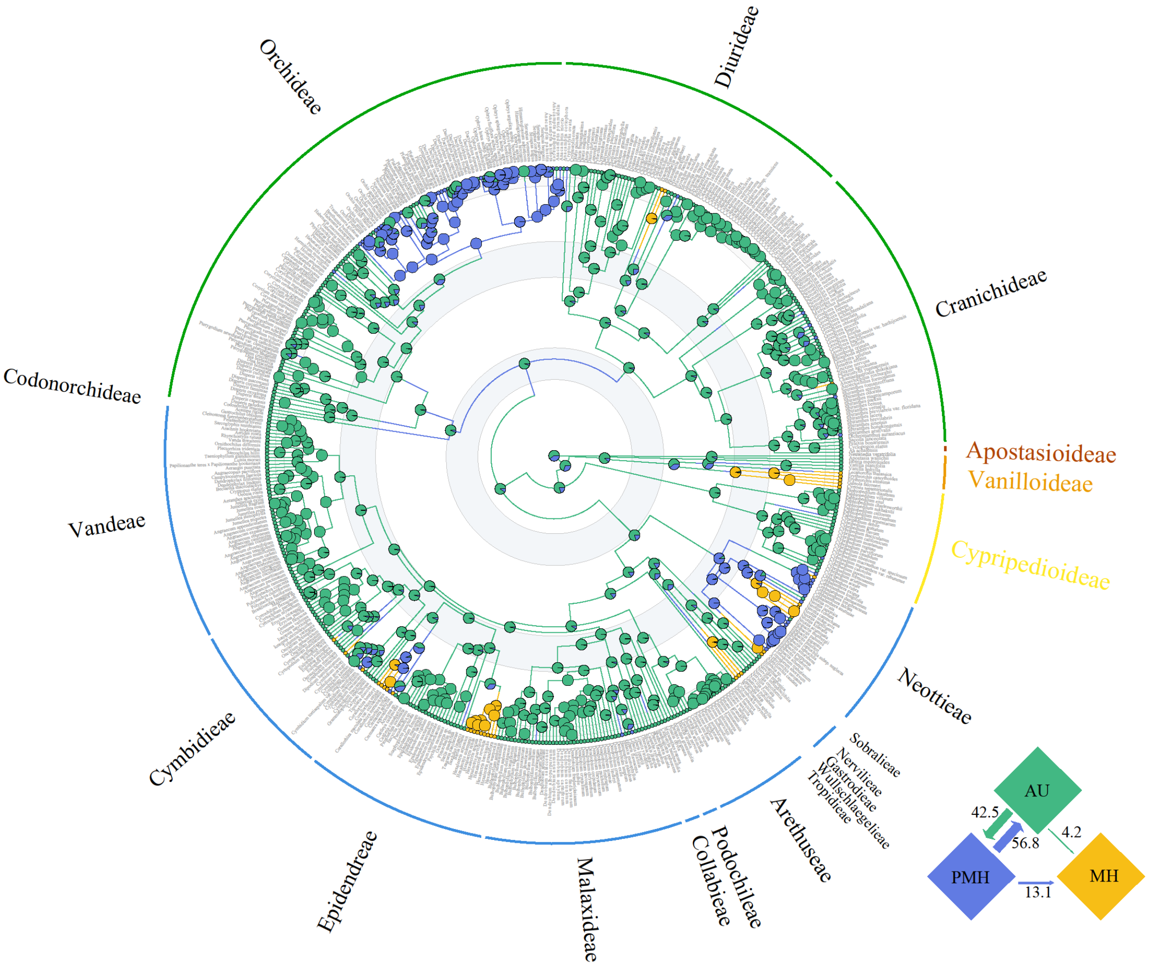

**Fig. S6 Ancestral state reconstruction of the trophic mode under a strict definition of partial mycoheterotrophy (PMH) using stochastic character mapping.** Using a strict definition of PMH (Supporting information Notes S1), orchid species are significantly enriched in both $^{13}C$ and $^{15}N$ compared with surrounding autotrophic vegetation. The number of transitions between trophic modes is visualized at the bottom right of the figure. The geological time scales are visualized by circles from upper Cretaceous (*c.* 90 – 66 MA), via Paleocene (*c.* 66 – 23 MA), and Neogene (*c.* 23 – 2.58 MA), to Quaternary (from 2.58 MA to the present)

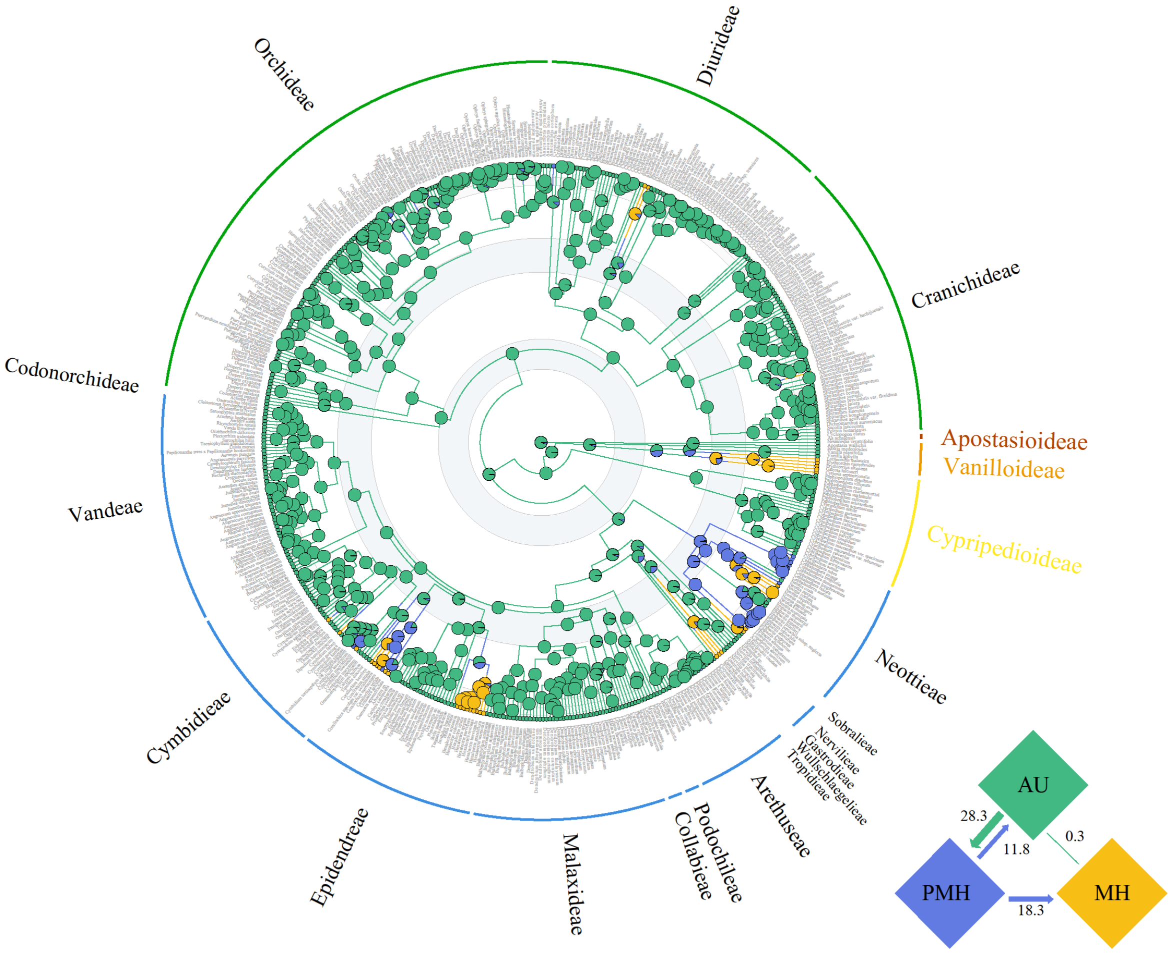

**Fig. S7 Changes of trophic mode and symbiotic association through time.** (a) The proportion of each trophic mode through time is sampled at 5 million year intervals on the orchid phylogeny with ancestral state reconstructions (Supporting Information Fig. S5). (b) The proportion of each symbiotic association is relative to the total number of branches at that particular point in time, sampled at 5 million year intervals on the orchid phylogeny with ancestral state reconstructions (Fig. 3). The geological time scales are visualized at the bottom from upper Cretaceous (*c.* 90 – 66 MA), via Paleocene (*c.* 66 – 23 MA), and Neogene (*c.* 23 – 2.58 MA), to Quaternary (from 2.58 MA to the present).

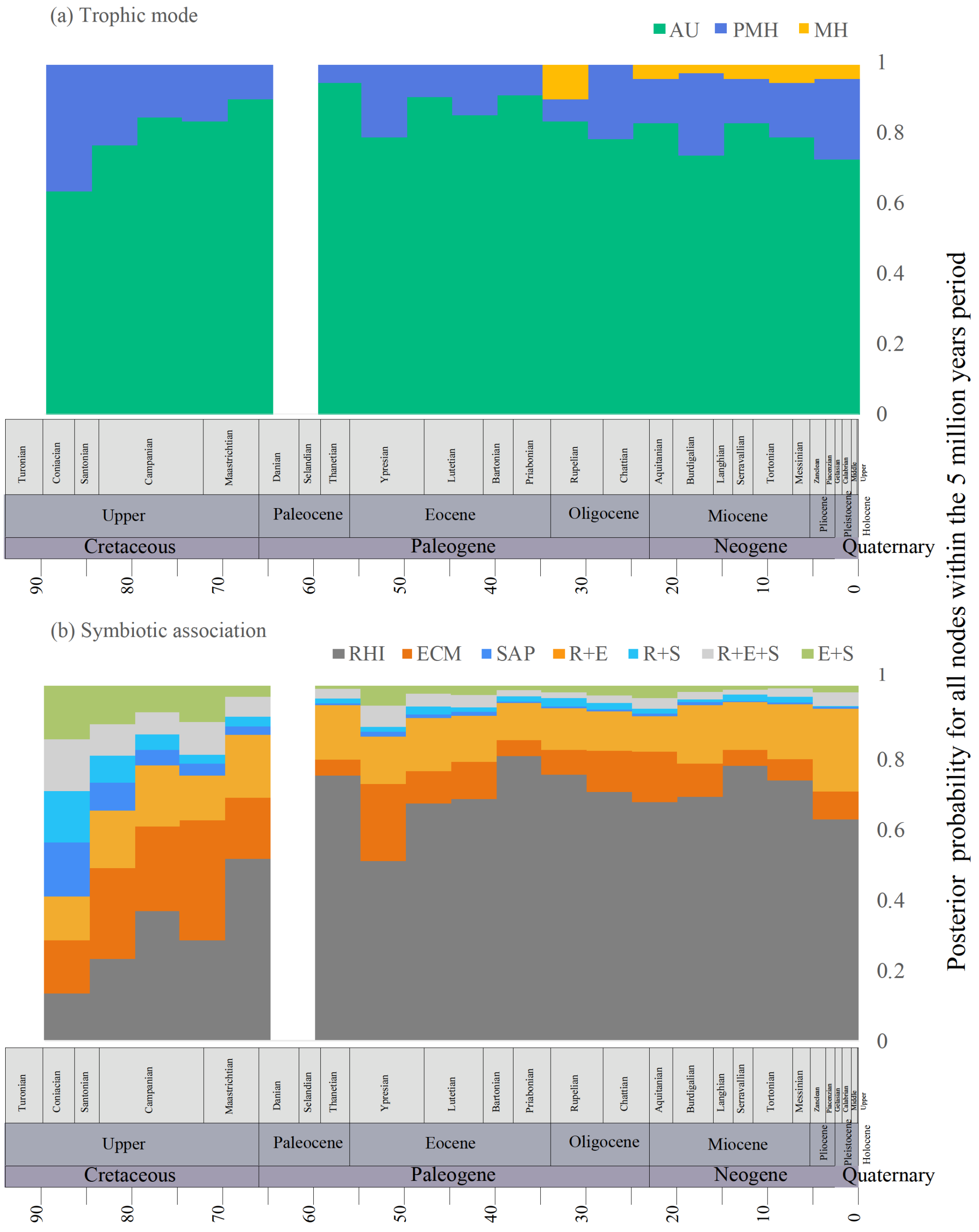

**Table S1** Orchid mycorrhiza dataset.

**Table S2** The list of orchid species for phylogenetic reconstruction and trait analyses.

**Table S3** Phylogenetic signals of symbiotic association and trophic mode using Pagel's lambda.

| Trait | Model test | | Phylogenetic signal (λ) |
|---|---|---|---|
| Symbiotic association | ARD model | log-likelihood = -481.80<br>AIC = 1077.60<br>AICc = 1091.321024 | 0.9 |
| Trophic mode | q(MH->PMH)=0; q(MH->AU)=0 | log-likelihood = -147.64<br>AIC = 305.29<br>AICc = 305.40 | 0.93 |

The ARD model of different rates in the state transition rate matrix (Q matrix) was conducted for symbiotic association and trophic mode. The symbiotic association represented by fungal lifestyle was classified as RHI, ECM, SAP, and their combinations. We specified a constrained model for trophic mode, which disallows any transition from full mycoheterotrophy (MH) to partial mycoheterotrophy (PMH) and autotrophy (AU), respectively, by setting their transition rate to zero. Pagel's lambda (λ) was used to evaluate the degree of phylogenetic signal. $\lambda = 0$ indicates no phylogenetic signal in the trait, indicating that the trait has evolved independently from the phylogenetic relationships. Conversely, $\lambda = 1$ indicates a strong phylogenetic signal, suggesting that the trait has evolved under a Brownian motion model of evolution.

**Table S4** Phylogenetic correlations between orchid trophic mode and symbiotic association using the Discrete Independent and Dependent models implemented in BayesTraits.

| Model Test | Independent Test: lnL | Dependent Test: lnL | Bayes Factor (BF) | Traits Correlation |
|---|---|---|---|---|
| Trophic mode ~ Symbiotic state | -304.56 | -275.25 | 58.62 | Very strong evidence |

The multistate discrete traits (trophic mode, symbiotic association) were separately converted into dummy states to fit the requirements of Discrete models. The trophic mode was classified as autotrophic (AU) and mycoheterotrophic (PHM and MH). The symbiotic association represented by fungal lifestyle was classified as rhizoctonia-like fungi (0-RHI) and non-rhizoctonia fungi (1-ECM, SAP, and all other combinations with RHI). The level of correlation was evaluated by a log Bayes Factor (BF). BF Interpretation: <2 Weak evidence; >2 Positive evidence; 5-10 Strong evidence; >10 Very strong evidence.

**Notes S1 Additional methodological details and results.** Detailed descriptions include fungal OTU clustering and taxonomic assignment, classification of trophic mode, orchid phylogeny reconstruction and divergence time estimation, correlated trait evolution, and hypothesis tests, as well as results of a parallel analysis investigating whether DNA sequencing techniques influence the fungal family composition.

## Supporting Information Notes S1

## Materials and Methods

### *Fungal OTU Clustering and Taxonomic Assignment*

To assign the taxonomic information of nearly 7000 fungal ITS sequences detected in orchid roots, we implemented "usearch_global" command with USEARCH v11 (Edgar, 2010) to blast against the UNITE local database (Abarenkov *et al.*, 2010) adopting a threshold of 97% identity. Because about 88% of all fungal sequences were assigned at the family level and 53% of all fungal sequences failed to be assigned to the genus or species level, we discarded all fungal sequences with taxonomic assignments above the family level only. Subsequently, seventy-two fungal families associated with only a single orchid species were also discarded since these represent autapomorphies that are not informative for ancestral state reconstructions (Yeates, 1992). Furthermore, we removed two fungal families not belonging to Basidiomycota and Ascomycota, resulting in 68 fungal families. We adopted a conservative strategy that we only kept families that contain putative orchid mycorrhizal fungi (Table 12.1 in Dearnaley *et al.*, 2012), resulting in a final set of 17 fungal families for further analyses.

### *Robustness to Sampling Uncertainty*

The fungal data in our dataset was produced by both Sanger and high-throughput sequencing. High-throughput sequencing (HTS) methods commonly produce millions of reads per run, and therefore possibly detect many more fungal taxa than traditional Sanger sequencing approaches. In our dataset, the fungal sequences of 69 orchid species were produced by HTS approaches either using Illumina or Roche 454 platforms. To assess the effect of the sequencing platform on the detected diversity of fungal families, we used USEARCH to blast fungal sequences excluding those produced by HTS against UNITE database. In addition, we

compared the detected fungal families of eleven orchid species for which data of both methods were available.

*Classification of the Trophic Mode*

The level of heterotrophy can vary across developmental stages (Bidartondo & Read, 2008; Těšitelová *et al.*, 2015; Waud *et al.*, 2017), thus we only considered the trophic state of a species at its adult stage. Orchid species were assigned to a trophic mode as follows: Species lacking visible chlorophyll were labeled as fully mycoheterotrophic. Green-leaved species were designated as autotrophic (initially mycoheterotrophic) unless the $^{13}C$ and $^{15}N$ data suggest that they are partially mycoheterotrophic at the adult stage. Despite the trophic state of species individuals may vary across habitats (Bidartondo *et al.*, 2004; Jacquemyn *et al.*, 2016; Duffy *et al.*, 2019), we assigned an orchid species as partially mycoheterotrophic when stable isotope enrichments of its individuals is confirmed by at least a single study. Species with significant enrichment in both $^{13}C$ and $^{15}N$ compared to surrounding autotrophic vegetation were classified as partially mycoheterotrophic (a strict definition of partial mycoheterotrophy). Species with enrichments in only $^{13}C$ or $^{15}N$ were considered as putative partially mycoheterotrophic (a relaxed definition of partial mycoheterotrophy). Since the $^{12}C/^{13}C$ and $^{14}N/^{15}N$ isotope ratios of only a few species have been investigated, mostly in temperate regions, and because C and N stable isotope signatures cannot detect low levels of mycoheterotrophy (Gebauer *et al.*, 2016; Schiebold *et al.*, 2018; Schweiger *et al.*, 2019), this approach is conservative and therefore may have underestimated the number of partially mycoheterotrophic species in our dataset.

*Orchid Phylogeny Reconstruction and Divergence Time Estimation*

After merging data of species with synonymous names using the "plantlist" R package (https://www.github.com/helixcn/plantlist) and removing species lacking DNA data, we kept a total of 498 orchid species for phylogenetic analysis. Based on Chase *et al.* (2015), we added data of 21 species to represent genera that lacked DNA accessions recorded in the dataset, 18 species belonging to tribes and subtribes unrecorded in our dataset, and two species to represent calibration nodes, thus resulting in a total of 539 species for phylogenetic reconstruction (Supporting Information Table S2). The DNA sequences of four universal markers (ITS, *matK*, *rbcL*, and *trnL-F*) of the selected orchids were downloaded from the NCBI GenBank database and aligned separately by MUSCLE (Edgar, 2004). We used

ModelTest-NG (Darriba *et al.*, 2020) to determine the substitution model for each alignment. The *GTR+I+G4* model was selected as the best substitution model for all partitions using the AICc.

To reconstruct a time-calibrated phylogeny of Orchidaceae, we performed a relaxed molecular clock analysis with BEAST v2.5 (Bouckaert *et al.*, 2019), constraining the two species *Apostasia wallichii* and *Neuwiedia veratrifolia*, belonging to Apostasioideae, as the outgroup, and tribes of orchids as monophyletic according to backbone trees in the references (Chase *et al.*, 2015; Givnish *et al.*, 2015; Chomicki *et al.*, 2015). Yule prior (Heled & Drummond, 2015) with a birth rate under a uniform prior, a relaxed clock lognormal model (Drummond *et al.*, 2006), and the estimated clock rate were chosen for the analysis. Three ingroup fossil calibration points were applied under a gamma distribution following Chomicki et al. (2015): (1) the monophyletic tribe Goodyerinae (15-20 Ma, offset = 17.5, SD = 5) (Ramirez *et al.*, 2007); (2) *Dendrobium* (20-23 Ma, offset = 20, SD = 4.5) (Conran *et al.*, 2009; Iles *et al.*, 2015); and (3) *Earina* (20-23 Ma, offset = 20, SD = 4.5) (Conran *et al.*, 2009; Iles *et al.*, 2015). Five parallel runs of Markov chain Monte Carlo (MCMC) searches (with a length of 100 million generations and sampling every 10,000 generations) were performed on CIPRES Science Gateway (http://www.phylo.org/). The effective sample size (ESS) of major traced parameters exceeded 200 (and all others exceeded 100) using a range of 20% ~ 70 % sampling trees as burn-in for parallel runs. We then calculated a majority-rule consensus over the retained trees.

*Correlated Trait Evolution*

To test for correlated evolution between trophic mode and symbiotic association, we implemented the Discrete Independent and Dependent models in BayesTraits V3. First, we converted all multistate traits into binary states to fit the requirements of the Discrete models. Trophic modes were recoded as mycoheterotrophic “MH” and non-mycoheterotrophic “non-MH” (AU + PMH). Likewise, the symbiotic state represented by fungal lifestyles were divided into “rhizoctonias” and “non-rhizoctonias” (ECM, SAP). Afterward, we ran $10^6$ iterations of reJUMP MCMC analyses (Pagel & Meade, 2006; Green, 1995) with BayesTraits. We used the “stones” command to approximate the marginal likelihood, running 100 stones for 10000 iterations. Finally, the log Bayes Factor (BF) was used to calculate the level of the correlation between each pair of discrete traits.

*Hypothesis Tests for Plausible Evolutionary Scenarios*

Based on the occurrence of coupled characters (Supporting Information Table S2), we identified five commonly observed multistate characters based on trophic mode and symbiotic association: 1) autotrophic orchids associating with rhizoctonia fungi (AU-R); 2) autotrophic orchids associating with ectomycorrhizal and/or saprotrophic fungi (AU-ES); 3) autotrophic orchids associating with rhizoctonia, ectomycorrhizal and/or saprotrophic fungi (AU-RES); 4) mycoheterotrophic orchids associating with rhizoctonia fungi, ectomycorrhizal and/or saprotrophic fungi (MH-RES), and 5) mycoheterotrophic orchids associating with ectomycorrhizal and/or saprotrophic fungi (MH-ES). To limit the number of free parameters in the Q matrix, which would have impeded convergence in our analysis, we put partially mycoheterotrophic states into the categories of fully mycoheterotrophic states and excluded the rare cases of combinations that do not fit the five states above. For example, three mycoheterotrophic species in the genera *Rhizanthella* and *Chamaegastrodia* that are associated with ectomycorrhizal members of the Ceratobasidiaceae were put into the MH-ES state because we treat Ceratobasidiaceae solely as 'rhizoctonias' in this study regardless of rare cases of ectomycorrhizal members in this family. In addition, the single instance of a putatively autotrophic species associating with saprotrophic fungi (*Cremastra appendiculata*) was categorized as the AU-ES state.

We tested specific paths of symbiotic shifts along which mycoheterotrophy has potentially evolved via a prerequisite of an intermediate symbiotic state. Therefore, we set constraints on the Q matrix and tested which state was best supported to be the intermediate state that enabled the evolution of mycoheterotrophy (see Diagram S1). The first model assumes that AU-R is the intermediate state just before the evolutionary transition to mycoheterotrophic states MH-RES and MH-ES. In this model, we disallowed direct transitions from AU-ES and AU-RES to the fully mycoheterotrophic states by setting their transition rates to zero. The second model assumes that AU-ES is the intermediate state for the evolution of mycoheterotrophy. In this model, we disallowed any transitions from AU-R and AU-RES to the fully mycoheterotrophic states. The third model assumes the AU-RES state as the intermediate state towards mycoheterotrophy by setting the transition rate of AU-R and AU-ES to MH-RES and MH-RES to zero. The fourth model is based on the previous model and represents a more specific path towards mycoheterotrophy by going through an obligate association with a combination of rhizoctonia, ectomycorrhizal and/or saprotrophic fungi, for

which the transition rate from AU-RES to MH-RES was also set to zero. While all four models confine the forward direction of transitions, the fifth model is identical to the fourth but also sets constraints on reverse transitions and assumes that transitions from the MH-ES state to the autotrophic states are impossible. Lastly, the final model only allowed that reversed transitions from mycoheterotrophic state to autotrophic state pass through an intermediate of a mixture of rhizoctonias, ectomycorrhizal, and/or saprotrophic fungi (MH-RES and AU-RES).

To compare models, we estimated the marginal likelihood of the free model where no constraints were set to the Q matrix. One constrained model disallowing specific transitions between states in the Q matrix that best fit the data will result in the marginal likelihood that differs most significantly from the less-constrained free model. We ran each 'MultiState' analysis in triplicate for $10^6$ generations and calculated the average marginal likelihood using a stepping stone sampler. Finally, we compared the constrained models with the free model by Bayesian information criterion (BIC), and the model with the lowest BIC values was preferred.

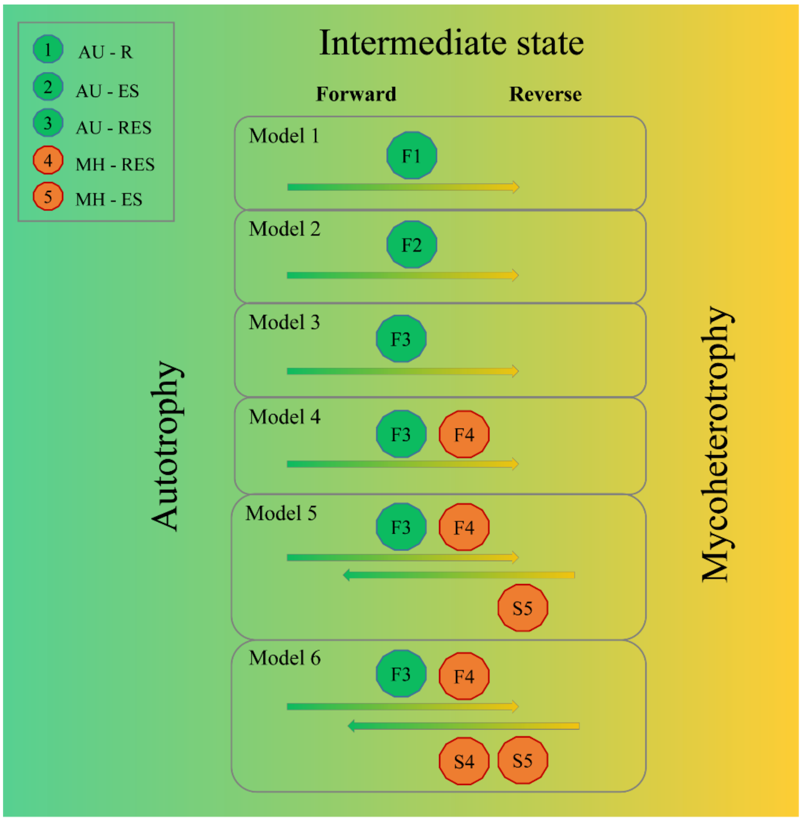


Diagram S1 Evolutionary models that constrain intermediate state(s) towards mycoheterotrophy.

## Results

*The Robustness of Our Analyses*

After removing fungal families that associate with only a single orchid, a total of 66 fungal families in Basidiomycota and Ascomycota were detected to present in 519 orchid species when reanalyzing the data without the HTS data (Supplementary Fig. S3a), which is similar with a set of 68 fungal families when analyzing all DNA sequences in our dataset (Supplementary Fig. S1). When comparing the fungal families associating with eleven orchid species between sequencing platforms, we found that the diversity of fungal families is slightly higher by Sanger sequencing than by HTS machines (Supplementary Fig. S3b). The slight bias in the diversity of fungal families could potentially relate to a larger number of studies adopting Sanger sequencing than the HTS method (Supplementary Fig. S3c).

## References

**Abarenkov K, Henrik Nilsson R, Larsson K-H, Alexander IJ, Eberhardt U, Erland S, Høiland K, Kjøller R, Larsson E, Pennanen T, *et al.* 2010**. The UNITE database for molecular identification of fungi - recent updates and future perspectives. *New Phytologist* **186**: 281–285.

**Bidartondo MI, Burghardt B, Gebauer G, Bruns TD, Read DJ**. **2004**. Changing partners in the dark: Isotopic and molecular evidence of ectomycorrhizal liaisons between forest orchids and trees. *Proceedings of the Royal Society of London. Series B: Biological Sciences* **271**: 1799–1806.

**Bidartondo MI, Read DJ**. **2008**. Fungal specificity bottlenecks during orchid germination and development. *Molecular Ecology* **17**: 3707–3716.

**Bouckaert R, Vaughan TG, Barido-Sottani J, Duchêne S, Fourment M, Gavryushkina A, Heled J, Jones G, Kühnert D, De Maio N, *et al.* 2019**. BEAST 2.5: An advanced software platform for Bayesian evolutionary analysis. *PLoS Computational Biology* **15**: e1006650.

**Chase MW, Cameron KM, Freudenstein J V, Pridgeon AM, Salazar G, Van Den Berg C, Schuiteman A**. **2015**. An updated classification of Orchidaceae. *Botanical Journal of the Linnean Society* **177**: 151–174.

**Chomicki G, Bidel LPR, Ming F, Coiro M, Zhang X, Wang Y, Baissac Y, Jay-Allemand C, Renner SS**. **2015**. The velamen protects photosynthetic orchid roots against UV-B damage, and a large dated phylogeny implies multiple gains and losses of this function during the Cenozoic. *New Phytologist* **205**: 1330–1341.

**Conran JG, Bannister JM, Lee DE**. **2009**. Earliest orchid macrofossils: Early Miocene *Dendrobium* and *Earina* (Orchidaceae: Epidendroideae) from New Zealand. *American Journal of Botany* **96**: 466–474.

**Darriba D, Posada D, Kozlov AM, Stamatakis A, Morel B, Flouri T**. 2020. ModelTest-NG: a new and scalable tool for the selection of DNA and protein evolutionary models. *Molecular Biology and Evolution* **37**: 291–294.

**Dearnaley JDW, Martos F, Selosse M-A**. **2012**. Orchid mycorrhizas: Molecular ecology, physiology, evolution and conservation aspects. In: *Fungal Associations*. Berlin, Heidelberg: Springer Berlin Heidelberg, 207–230.

**Drummond AJ, Ho SYW, Phillips MJ, Rambaut A**. **2006**. Relaxed phylogenetics and dating with confidence. *PLoS Biology* **4**: e88.

**Duffy KJ, Waud M, Schatz B, Petanidou T, Jacquemyn H**. **2019**. Latitudinal variation in mycorrhizal diversity associated with a European orchid. *Journal of Biogeography* **46**: 968–980.

**Edgar RC**. **2004**. MUSCLE: Multiple sequence alignment with high accuracy and high throughput. *Nucleic Acids Research* **32**: 1792–1797.

**Edgar RC**. **2010**. Search and clustering orders of magnitude faster than BLAST. *Bioinformatics* **26**: 2460–2461.

**Gebauer G, Preiss K, Gebauer AC**. **2016**. Partial mycoheterotrophy is more widespread among orchids than previously assumed. *New Phytologist* **211**: 11–15.

**Givnish TJ, Spalink D, Ames M, Lyon SP, Hunter SJ, Zuluaga A, Iles WJD, Clements MA, Arroyo MTK, Leebens-Mack J, *et al.* 2015**. Orchid phylogenomics and multiple drivers of their extraordinary diversification. *Proceedings of the Royal Society B: Biological Sciences* **282**: 20151553.

**Green PJ**. **1995**. Reversible jump Markov chain Monte Carlo computation and Bayesian model determination. *Biometrika* **82**: 711–732.

**Heled J, Drummond AJ**. **2015**. Calibrated birth-death phylogenetic time-tree priors for Bayesian inference. *Systematic Biology* **64**: 369–383.

**Iles WJD, Smith SY, Gandolfo MA, Graham SW**. **2015**. Monocot fossils suitable for molecular dating analyses. *Botanical Journal of the Linnean Society* **178**: 346–374.

**Jacquemyn H, Waud M, Lievens B, Brys R**. **2016**. Differences in mycorrhizal communities between *Epipactis palustris*, *E. helleborine* and its presumed sister species *E. neerlandica*. *Annals of Botany* **118**: 105–114.

**Pagel M, Meade A**. **2006**. Bayesian analysis of correlated evolution of discrete characters by Reversible-Jump Markov Chain Monte Carlo. *The American Naturalist* **167**: 808–825.

**Ramirez SR, Gravendeel B, Singer RB, Marshall CR, Pierce NE**. **2007**. Dating the origin of the Orchidaceae from a fossil orchid with its pollinator. *Nature* **448**: 1042–1045.

**Schiebold JMI, Bidartondo MI, Lenhard F, Makiola A, Gebauer G**. **2018**. Exploiting mycorrhizas in broad daylight: Partial mycoheterotrophy is a common nutritional strategy in meadow orchids. *Journal of Ecology* **106**: 168–178.

**Schweiger JMI, Kemnade C, Bidartondo MI, Gebauer G**. **2019**. Light limitation and partial mycoheterotrophy in rhizoctonia-associated orchids. *Oecologia* **189**: 375–383.

**Těšitelová T, Kotilínek M, Jersáková J, Joly F-X, Košnar J, Tatarenko I, Selosse M-A**. **2015**. Two widespread green *Neottia* species (Orchidaceae) show mycorrhizal preference for Sebacinales in various habitats and ontogenetic stages. *Molecular Ecology* **24**: 1122–1134.

**Waud M, Brys R, Van Landuyt W, Lievens B, Jacquemyn H**. **2017**. Mycorrhizal specificity does not limit the distribution of an endangered orchid species. *Molecular Ecology* **26**: 1687–1701.

**Yeates D**. **1992**. Why remove autapomorphies? *Cladistics* **8**: 387–389.

**Yu G**. **2020**. Using ggtree to visualize data on tree-like structures. *Current Protocols in Bioinformatics* **69**: e96.
